# Developing orientation maps using realistic patterns of lateral connectivity

**DOI:** 10.1186/1471-2202-14-S1-P21

**Published:** 2013-07-08

**Authors:** Philipp Rudiger, Judith S Law, Jan Antolik, James Bednar

**Affiliations:** 1 Institute for Adaptive and Neural Computation, University of Edinburgh, Edinburgh, EH8 9AB, UK; 2 Unit of Information and Complexity (UNIC), CNRS, Gif-sur-Yvette, 91198, France

## 

While developmental models have been very successful in replicating the main features of experimentally observed topographic maps in the primary visual cortex (V1), they have relied on several unrealistic assumptions. These models are typically variants of the self-organizing map model [1], and almost universally assume "Mexican-hat" lateral connectivity in V1, with short-range excitatory and longer-range inhibitory connections. Experimental data is in direct conflict with this assumption, with anatomical tracing studies showing that neurons making long-range connections are excitatory [2,3]. A variety of electrophysiological and psychophysical studies also suggest both excitatory and inhibitory effects at long ranges, depending on experimental conditions. The current consensus is that the actual pattern of connectivity consists of long-range excitation leading to di-synaptic inhibition via local inhibitory interneurons [2-4]. The resulting aggregate circuit has an overall inhibitory effect when the excitatory drive to local inhibitory synapses is large enough. In principle, the behavior of this circuit at high input contrasts may therefore mimic the Mexican-hat profile of these earlier model, while potentially exhibiting more realistic contrast dependent behavior.

We present a rate-based model of simple-cell development that robustly self-organizes into biologically realistic orientation maps on the basis of this experimentally determined connectivity. The model is built using the Topographica simulator [5], and consists of a number of sheets of units representing the retinal photoreceptors, RGC/LGN cells, and individual populations of excitatory and inhibitory V1 neurons. The receptive field weights, initialized randomly within a Gaussian envelope, are adjusted through Hebbian learning with divisive normalization in response to activity driven by 20,000 consecutive input patterns (either natural images or artificial patterns). We show that development of realistic maps is robust, primarily due to homeostatic mechanisms in V1 and divisive contrast-gain control in the RGC/LGN layer.

The model demonstrates that the experimentally established connectivity framework can lead to orderly map development and can replicate many of the contextual and contrast dependent effects observed in adult V1. This work looks at how Mexican-hat connectivity arises from the overall network interactions at high contrast and how it adjusts at lower contrasts. Further, it demonstrates clearly how patchy long-range connectivity between iso-orientation domains emerges, and the role it plays in modulating V1 activity. In doing so, the model provides a clear link between topographic map formation, the development of the underlying connectivity, and the perceptual consequences of this circuitry, including contrast-dependent size-tuning shifts and the early stages of more complex effects like pop-out and contour completion.

In future, this work will help us to complete our understanding of the V1 circuit by adding feedback mechanisms or selectively modulating specific connections to model the effects of different neuromodulators. Additionally, the results may be used to provide realistic connectivity patterns for large scale spiking models, which often struggle to adequately constrain their connectivity. Overall, this model demonstrates for the first time that it is possible to robustly develop biologically plausible orientation maps on the basis of realistic connectivity, accounting for various surround modulation effects and providing a solid basis for future models of V1.
